# Evaluating the Effectiveness of Community-Delivered Hearing Rehabilitation and Health Education Intervention on Social Isolation and Functioning Among Chinese Adults With Hearing Impairment: Protocol for Randomized Controlled Trial

**DOI:** 10.2196/64115

**Published:** 2025-04-28

**Authors:** Jiamin Gao, Yuying Zhang, Xiaqing Jiang, Zhenjing Fu, Haochen Jiang

**Affiliations:** 1 School of Sociology Beijing Normal University Beijing China; 2 School of Government Beijing Normal University Beijing China; 3 School of Economics and Management Fuzhou University Fuzhou China

**Keywords:** hearing impairment, social isolation, functioning, community-delivered hearing interventions, randomized controlled trial

## Abstract

**Background:**

Hearing impairment (HI) is a common sensory deficit with considerable impacts on social well-being (SWB) in adults. Evidence on the effectiveness of auditory rehabilitation and hearing health education in the social domain of health for individuals with HI is scarce.

**Objective:**

This study aims to test the feasibility and efficacy of providing free hearing aids or a combined offline and online hearing health education intervention on social isolation and functioning among Chinese adults with HI.

**Methods:**

This study is a 3-arm, single-blinded, randomized controlled trial (RCT) with a follow-up at 24 months after the baseline study. A total of 435 participants aged 18 years and older with some degree of HI will be recruited and randomly assigned to 2 intervention groups and 1 control group. Free hearing-aid provision, as well as a hearing health education program that is combined with online and offline lessons, will be implemented in 2 intervention groups, respectively. The control group will not receive any intervention. The primary outcomes include social isolation and functioning in society. The secondary outcomes include social engagement, a sense of mastery, self-efficacy, psychological resilience, chronic diseases, life satisfaction, hearing health literacy, and hearing care usage.

**Results:**

Participants were recruited for hearing tests in September 2022, during which baseline results were collected through in-person interviews. Follow-up interviews were conducted in September 2024. The primary analysis will use ANOVA, linear mixed-effects modeling, structural equation modeling, and cost-effectiveness analysis.

**Conclusions:**

The findings of this study will provide evidence for the impact and cost-effectiveness of a community-based auditory or hearing health education intervention on SWB among Chinese adults with HI, which may contribute to promoting hearing health and reducing adverse health consequences in an aging society.

**Trial Registration:**

Chinese Clinical Trial Registry ChiCTR2200062148; https://www.chictr.org.cn/showproj.html?proj=174741

**International Registered Report Identifier (IRRID):**

DERR1-10.2196/64115

## Introduction

### Background

Hearing impairment (HI) significantly impacts the health and well-being of adults, especially middle-aged and older individuals. Globally, approximately 1.57 billion people had some degree of HI in 2019, with nearly 30% suffering from disabling HI and the majority of whom were aged ≥45 years [1]. HI is associated with various negative consequences, including declines in physical and cognitive functions, increased depression and anxiety, and heightened risks of frailty, hospitalization, and mortality [2-7]. Unaddressed HI often leads to significant withdrawal from the social domain of life and poor performance of functioning in society.

Social well-being (SWB) not only encompasses one’s ability to establish healthy relationships and engage meaningfully with social networks but also represents a subjective evaluation of personal life circumstances and functioning in society [8]. The World Health Organization (WHO) identifies SWB as a crucial element for overall health and has prioritized it worldwide to enhance social connections to fight against social isolation, which is often regarded as a common reflection of poor SWB [9]. Social isolation, defined as an objective absence or paucity of contacts and interactions between a person or within a social network [10,11], is associated with HI and is often considered a potential pathway to adverse health outcomes related to HI [12]. Communication difficulties or mental fatigue attributed to HI, as well as the increased cognitive load required to compensate for auditory deficits often discourage social interactions among those with HI [13-15].

Compared to social isolation, functioning in society is a broader and more comprehensive measure for assessing one’s capacity to interact with others, contribute to various societal roles, and actively engage in meaningful activities, including but not limited to work, education, family life, and community involvement. The International Classification of Functioning, Disability and Health (ICF), provides a framework for assessing functioning in society across three levels: the body or body part, the whole person, and the whole person in a social context, corresponding to its definition of functioning encompassing all body functions, activities, and participation [16]. Activity limitations and participation restrictions, which measure functions from both individual and societal perspectives, refer to a person’s capacity in a standard environment and performance in a normal environment under healthy conditions. The validation of the ICF application for adults with HI and in audiological or rehabilitation research has been examined internationally [17,18], demonstrating a positive association between hearing loss and decline in mobility, self-care, or the ability to get along with others [2,19,20]. Despite evidence supporting HI as a modifiable risk factor for social health, the efficacy of HI interventions in enhancing SWB requires further research [21,22]. Previous studies either have shown mixed results with little consistency in the significance and magnitude of the effectiveness of hearing intervention on social isolation or functioning [13,23-25], or used small sample sizes, and nonrandomized controlled trials to identify the causal relationships between hearing intervention and improvements in SWB [13,26,27]. Furthermore, few interventional studies have further discussed and tested the possible mechanistic pathway through psychosocial or supportive factors, and evidence regarding middle-income countries is scarce.

### Conceptual Framework and Interventions

Both the ICF and stress-buffering models provide insights into how adult-onset HI affects SWB and the effectiveness of auditory interventions. The ICF describes the bidirectional association among impairments, activity limitations, and participation restrictions. Besides, functioning can be viewed as the outcome of interactions between health conditions and contextual factors, which include external environmental factors (eg, supportive environment or social attitudes) and internal personal factors (eg, coping style and psychological resources) [16]. The ICF reveals that the ideal interventions should consider a holistic approach combining physical, personal, and environmental factors. The stress-buffering model, derived from the stress process paradigm, posits that social and coping resources can buffer (mediate or moderate) the impacts of stressors such as HI on health and well-being by altering one’s appraisal and coping behaviors toward a stressor or threat [28,29]. Empirical studies support the potential psychological resilience to mediate the relationship between functional HI and SWB [14].

Intervention strategies typically focus on individual or environmental approaches. Individual strategies such as audiological training, skill development, and health education aim to use residual hearing through individually tailored intervention and improve communication and coping skills for persons with HI or their communication partners (CPs). Skill development and health education are person-centered approaches that can be widely promoted and implemented at the community level. Either an appropriate use of hearing aids or assistive devices to compensate for auditory loss, the development of self-management skills for HI, or the application of positive communications, are often considered potential effective skill-developing interventions. Educative interventions in the hearing rehabilitation context empower adults with HI and their CPs to adapt to hearing deficits and improve help-seeking behaviors. Hearing health education (HHE) involves the dissemination of knowledge about the nature of hearing and hearing loss, communication strategies, the promotion of hearing devices or assistive technologies use, and the enhancement of psychosocial resources. Environmental strategies center on enhancing support systems within micro and meso environments, such as family and community, by providing information and guidance to the significant others and helping them better understand the challenges faced by adults with HI, as well as fostering active communication skills, awareness, and access for hearing health care.

**Figure 1:**
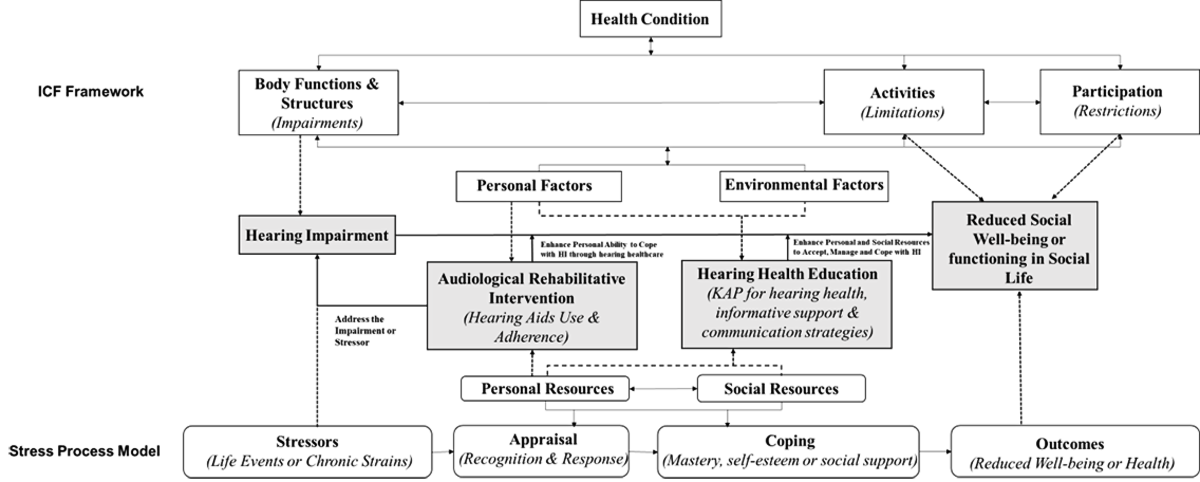
The theoretical framework of Hearing Impairment and Social Outcomes Evaluation in Adults developed based on the International Classification of Functioning, Disability and Health and stress-buffering model. HI: hearing impairment; ICF: International Classification of Functioning, Disability and Health; KAP: Knowledge, Attitude, and Practice Theory.

In response to the WHO’s guidelines on integrated person-centered ear and hearing care (IPC-EHC), we initiated the HISEA (Hearing Impairment and Social Outcomes Evaluation in Adults) project to conduct hearing interventions that targeted the social isolation and functioning of HI persons in Guangdong Province, China. This community-delivered intervention aligns with the China Disability Action Plan (2021-2025), which emphasizes the importance of raising public awareness of hearing prevention and care through health education as well as treating HI through rehabilitative services. Figure 1 presents a diagram illustrating how the interventions developed in HISEA align with the ICF and stress-buffering framework.

### Study Aims and Hypotheses

This full-scale randomized controlled trial (RCT) aims to evaluate the feasibility and efficacy of a community-delivered hearing aid provision and HHE program in improving social health among hearing-impaired adults in China. The key outcomes are social isolation and societal functioning. In addition, it seeks to determine if psychosocial factors and social support buffer the HI-SWB linkage as well as the effects of hearing interventions on social health.

We hypothesize there will be positive effects after the long-term interventions of HAU and HHE on hearing-impaired older adults’ social health, and strengthened psychological well-being could be potential pathways.

## Methods

### Overview

This protocol was developed on the basis of the theoretical framework in Figure 1 and is reported according to the Standard Protocol Items for Interventional Trials (SPIRIT) [30,31]. A peer review report can be found in Multimedia Appendix 1, and a SPIRIT checklist can be found in Multimedia Appendix 1.

### Feasibility Study

Before the initiation of the full-scale RCT, a feasibility study was undertaken. This study involved a comprehensive review and evaluation of the overall implementation plan by community social workers and audiologists to ascertain its feasibility from a health care professional perspective. In addition, a cohort of 28 adults with HI was recruited from Zhaoqing City, Guangdong Province, to conduct hearing checks and baseline interviews. During this process, we evaluated the participants’ willingness to engage in the study, confirmed their comprehension of the survey questionnaires, and assessed their readiness to participate in health education initiatives or to wear hearing aids over a long period, should they be assigned to the treatment groups. We made some adjustments to the questionnaire based on the feedback from the respondents. The preliminary results validated the feasibility of our intervention study.

### Study Design and Setting

A three-arm, single-blinded RCT with a 24-month follow-up period will be conducted. A total of 435 eligible participants were randomly assigned in a 1:1:1 ratio to a control group, a hearing aid use (HAU) group, or an HHE group. The HAU group will receive hearing aids and follow-up instructions. Due to budget constraints, the audiological rehabilitation intervention predominantly used hearing aids provided by the Audiology Department of the Guangdong Provincial Cultural Sports and Rehabilitation Assistive Devices Center for People with Disabilities (GPSRC), which is affiliated with the China Disabled Persons’ Federation (CDPF). Individuals diagnosed with disabling HI and classified as persons with hearing disability are eligible to apply for complimentary hearing aids and fitting services from the local CDPF departments. Since the number of adults with HI who were eligible for hearing-aid fitting was limited to one site at the time of the baseline survey and intervention, a total of 139 persons with disabled HI were randomly selected from 4 sites with similar socioeconomic and cultural conditions. The selection was based on the list provided by GPSRC, which maintains records of hearing-disabled persons in Guangdong Province. The HHE group will undergo both offline and online educational interventions, while no interventions are planned for the control group that is similar to a natural setting. A sample of 278 adults diagnosed with some degree of HI was randomly selected and assigned to the HHE group and control group from Hualong County in Guangzhou, Guangdong, based on its roster. For the HAU group, participants were prescribed hearing aids at baseline (T0) and received reinstruction on the use of hearing aids 12 months after T0. For the HHE group, participants were required to participate in an offline hearing education at T0, followed by an online educational intervention at 6 months after T0, and an offline information intervention at 12 months after T0. The primary outcomes are social isolation and functioning, assessed at baseline and 24 months.

### Participants

Eligible participants are adults aged ≥18 years with adult-onset audiometric HI, without significant cognitive impairments or neurological conditions. Participants are prescreened via telephone and in-person visits conducted by community workers and confirmed with some degree of HI through audiometric assessment by professional audiologists. The eligibility criteria are mentioned in Textbox 1.

**Inclusion and exclusion criteria.**

**Inclusion criteria**

- Age (18 years or older): individuals aged 18 years at the time of recruitment are eligible for the trial. This age range meets the needs of this study by targeting adult-onset hearing impairment (HI). Besides, given that adult-onset HI is more prevalent in middle-aged and older adults, individuals who develop hearing loss after the age of 55 years are preferentially included.
- Residency: community-dwelling within the sites for the duration of the study.
- Audiometric hearing impairment: participants must have adult-onset audiometric HI with a 4-frequency (0.5, 1, 2, and 4 kHz) PTA hearing threshold ≥21 dB hearing level (HL) and ≤90 dB HL in a better hearing ear (ranging from mild, moderate, severe to profound hearing loss according to the World Health Organization (WHO) 2021 grades of hearing loss [32]). These grades of hearing loss indicate the level at which individuals are likely to benefit from the use of hearing aids or other amplified devices.
- No significant diagnosed or self-reported history of dementia or neurological impairments.
- Communication: Fluent in Mandarin or Cantonese with basic word recognition skills.
- Willing to participate, be randomized, and adhere to the protocol: Participants must be willing and able to consent to engage in the study, be willing to participate in the interventions, and commit to adhering to the study protocol until the end of the trial (24 months after the baseline survey).

**Exclusion criteria**

- Profound, complete, or total hearing loss or deafness: pure tone audiometry (PTA) at 4 frequencies for better ear of >90 dB HL, who are suggested to face challenges in effectively participating in the intervention setting [33].
- Significant ongoing ear-related diseases, medical contraindications to the use of hearing aids, or permanent conductive HI.
- Recent use of hearing aids or other listening devices: to assess the net effect of hearing interventions, participants should not be receiving any treatment for the HI already.
- Previous dementia diagnosis and self-reported mental health problems: self-reported diagnoses of dementia or memory-related diseases, and those who required a proxy to provide informed consent and answer questions due to cognitive problems or other mental health issues are excluded.
- Unwillingness to wear hearing aids regularly.
- Visual impairment: participants who are unable to see well enough (even with correction) to read a brochure or watch an online video are excluded.
- Unable or unwilling to provide written informed consent.

### Sample Size Calculation and Recruitment

Sample size calculations are based on the expected effects of the hearing aid intervention on the primary outcome: an individual’s functioning in society measured by the World Health Organization Disability Assessment Schedule 2.0 (WHODAS 2.0), which was developed within the ICF conceptual framework [34]. We estimate that a sample size of 222 (111 participants in each group) provides 85% statistical power with a 5% significance level to detect a standardized effect size of 0.404 for the difference between the HAU group and the control group in the mean change from baseline in the functioning score at the 24-month follow-up [23]. Since there is little evidence evaluating the effects of HAU on social isolation using the Lubben Social Network Scale (LSNS), or the effectiveness of HHE on functioning and social isolation, we will assign the HHE group the same sample size as the HAU group and the control group. To account for a 20% drop-in (uptake of hearing aids in the control group) and dropout (discontinuation of HAU in the hearing intervention group) rate, a total of 435 individuals are included. This comprises 121 individuals in the HAU group, 158 in the HHE group, and 156 in the control group, respectively. Recruitment activities are conducted in communities and villages (n=8) from Guangzhou, Shanwei, Chaozhou, and other areas in Guangdong Province, China. The recruitment of participants commenced on September 1, 2022, and was completed on September 31, 2022. Oral informed consent is obtained through prescreening, and written informed consent is presented by the investigator to the participant before the hearing screening. Hearing screening and assessment will be undertaken by an audiologist following a standardized procedure (Table 1).

**Table 1:** Schedule of enrollment, interventions, and assessments.

Time point				Study period																												
				Enrollment T_1_						Allocation T_0_					Postallocation T_1_							Postallocation T_2_						Close-out T_4_				
				–30 to –1 Day						Day 0					6 months							12 months						24 months				
				T^a^		T^b^		C^c^		T	T		C		T		T		C		T			T		C	T			T		C
Enrollment																																
Eligibility screen				✓		✓		✓																								
Informed consent				✓		✓		✓																								
Demographics				✓		✓		✓																								
Health history				✓		✓		✓																								
Visual status				✓		✓		✓																								
Allocation										✓	✓		✓																			
Interventions																																
Hearing aids provision										✓											✓						✓					
Hearing health education											✓						✓							✓						✓		
Assessments																																
Sociodemographics										✓	✓		✓														✓			✓		✓
Hearing outcomes																																
Pure-tone audiometry										✓	✓		✓																			
HHIA^d^ score										✓	✓		✓														✓			✓		✓
Self-reported hearing										✓	✓		✓														✓			✓		✓
IOI-HA^e^ score																											✓					
Hearing-related health literary										✓	✓		✓														✓			✓		✓
Communication ability											✓		✓														✓			✓		✓
SWB^f^ (primary outcome)																																
LSNS-6^g^ score										✓	✓		✓		✓												✓			✓		✓
WHODAS 2.0^h^ score										✓	✓		✓		✓												✓			✓		✓
ISE^i^ score										✓	✓		✓		✓												✓			✓		✓
SEAQ^j^ score										✓	✓		✓		✓												✓			✓		✓
Secondary outcomes																																
GSE^k^ score										✓	✓		✓														✓			✓		✓
PMS^l^ score										✓	✓		✓														✓			✓		✓
CD-RISC^m^ score										✓	✓		✓														✓			✓		✓
EQ-5D-5L score										✓	✓		✓														✓			✓		✓
SWLS^n^ score										✓	✓		✓														✓			✓		✓
Covariates										✓	✓		✓														✓			✓		✓
Lifestyle behaviors										✓	✓		✓														✓			✓		✓
Chronic diseases										✓	✓		✓														✓			✓		✓

^a^T: hearing aid intervention group.

^b^T: hearing health education intervention group.

^c^C: control group.

^d^HHIA: Hearing Handicap Inventory Screening Questionnaire for Adults.

^e^IOI-HA: International Outcome Inventory for Hearing Aids.

^f^SWB: social well-being.

^g^LSNS-6: Lubben Social Network Scale.

^h^WHODAS 2.0: World Health Organization Disability Assessment Schedule 2.0.

^i^ISE: Index of Social Engagement.

^j^SEAQ: Social Engagement and Activities Questionnaire.

^k^GSE: General Self-Efficacy Scale.

^l^PMS: Pearlin Mastery Scale.

^m^CD-RISC: Connor-Davidson Resilience Scale.

^n^SWLS: Satisfaction with Life Scale.

### Randomization, Allocation, and Masking

After confirming eligibility and informed consent, participants are randomly stratified by HI severity and the field site to ensure a balanced distribution of participants. Randomization is performed by an independent statistician who is not involved in data collection, management, or analysis using a computer-generated random number sequence to assign participants to either the treatment or control group [35]. Allocated interventions will not be modified unless participants voluntarily withdraw for personal reasons. Due to the nature of the interventions, complete blinding of participants and facilitators is not feasible. However, we minimize the potential biases by blinding participants to the study hypothesis, using standardized protocols for training and tools for measurement, and masking field staff to allocation details.

### Study Interventions

The community-delivered interventions are developed based on the conceptual framework of ICF and the stress-buffering model. The HAU group mainly targets addressing HI as a stressor or physical impairment and improving personal resources for managing HI. The HHE group aims to eliminate or minimize the social effects of HI by enhancing awareness, acceptance, adaptation, and support for HI through personal and social resources among adults with HI.

#### Hearing Rehabilitation Intervention: Hearing Aid Use Group

Participants in the hearing rehabilitation intervention treatment group are fitted with bilateral or unilateral hearing aids. The hearing aids are fitted to participants in accordance with the protocol established by the hearing aid manufacturer and the guidelines launched by CDPF with professional audiologists. Instructions on the usage of hearing aids, the proper insertion of the aids and batteries, and a comprehensive, step-by-step guide on their usage and maintenance, are also provided. Materials for self-management and adaptation strategies are also explained and provided after the fitting services. To improve hearing aid compliance, support is offered after fitting (1 month later) to ensure that each participant is progressing with their hearing aids. In addition, a WeChat (Tencent) group has been established for HAU participants and audiologists to address sporadic needs from hearing aid users, such as battery replacements, cleaning, or proper storage of hearing aids. Unscheduled interim visits are necessary for troubleshooting or repairing malfunctioning hearing aids or addressing other related issues if the problem cannot be resolved through online or telephone communication. Postintervention reinstruction on the use of hearing aids is conducted 12 months after the fitting through telephone communication and the dissemination of digital materials via the WeChat group.

#### The HHE Intervention

The HHE intervention group is developed based on the Health Education Services Standard of the National Basic Public Health Service Standard (third edition), a national guideline issued by the National Health Commission of the People’s Republic of China for guiding health education for community-dwelling residents [36]. It targets participants with HI, their CPs or family members, community residents, and hearing health–related community workers (ie, social workers, local staff of CDPF, and civil affairs). The objectives of HHE include improving awareness, knowledge, and literacy of hearing health among individuals, families, and communities, developing communication strategies and skills for adults with HI and their CPs or family members, as well as creating a supportive family and community environment that promotes social inclusion and accessibility to hearing health care services.

The HHE intervention consists of three parts. Part A is an interactive, face-to-face session consisting of a hearing health lesson and counseling for participants with HHE and community workers. Brochures for participants and their CPs or family members, and posters for community health education boards are provided. The hearing health lesson is taught by the program coordinators with a written lesson plan and prepared with easily accessible teaching materials, such as paper-based visual aids or slides. Part B is an internet-based educational section that aims to reinforce the content covered in Part A through the use of the WeChat public account. It was conducted 6 months after the baseline survey targeted adults with HI and their CPs. Part B consists of a 2-week online information support program delivering knowledge on hearing health and its consequences, positive skills and strategies for interacting with adults with HI, hearing prevention and rehabilitation guidance, and social support for adults with HI through textual, image, and video formats. All interventional-related information is disseminated through the WeChat public account registered by the research team. To ensure the accessibility and reach of online information support, the research team set up a check-in task and regularly reminded the procedure. Part C is an offline information provision that was conducted 12 months after the baseline survey with the purpose of reinforcing content in Part A and Part B. Hearing health educational pamphlets are designed for all the intervention objectives and are delivered to them accordingly. The content presented in the lessons, brochures, posters, a WeChat public account, and pamphlets is based on and adapted from the WHO Hearing Report (2021) and other relevant publications [37,38]. During the design phase of the intervention, the HHE intervention settings were discussed and deliberated with experienced audiologists, community workers, and community-dwelling adults with HI who would not participate in the study. It is deemed acceptable for adults with HHE and their CPs in the community.

### Control Group

No interventions are provided for the control group considering the current situation of the HI rehabilitation:

Firstly, the prevalence of hearing aid usage in China is notably low, and there is a lack of health care awareness among rural older adults experiencing HI. A population-based survey carried out between 2014 and 2015 across 4 provinces in China, including Guangdong, revealed that the estimated prevalence of hearing aid acquisition among rural older adults with hearing loss is 4.2%. The reasons for not having a hearing aid within this group included “not understanding its function,” “not being able to afford it,” and “not needing it” [39]. According to the China Health and Retirement Longitudinal Study 2018 data, only 3% of older adults in rural areas with subjective hearing loss self-reported experience of using hearing aids [40]. Both sets of data indicate that they resonate with the natural setting for the control group.

Secondly, the policy context in Guangdong Province requires the attainment of a disability status qualification before the application for government-subsidized hearing aids, potentially discouraging older adults with HI from initiating the application process. The government offers subsidized hearing aids at institutions specifically established to provide services related to assistive devices for individuals with hearing disabilities. However, it is essential for these individuals to actively secure an official assessment to obtain a disability certificate, which is a prerequisite for applying for subsidized hearing aids. The process of obtaining disability status may be associated with social stigma, which could inhibit individuals from applying despite the availability of government subsidies. Therefore, the number of disabled individuals seeking these hearing aids remains relatively low.

Considering these 2 points, we have opted not to intervene with the older adults in the control group. Participants who are eligible for the control group are required to engage in both the baseline and follow-up surveys.

### Outcome Measures

This study investigates the 24-month impact of HAU and HHE interventions on social isolation, functioning, and engagement among adults with HI and further explores the buffering role of psychosocial factors in these outcomes. The detailed outcomes are listed in Textbox 2.

**Outcome measures.**

**Primary outcome measures**

- Social isolation, assessed by the abbreviated 6-item Chinese version of the Lubben Social Network Scale (LSNS-6), which has been recognized as a good tool to screen for social isolation among community-dwelling adults by several cross-national and cross-cultural validation studies [41,42]. The LSNS-6 evaluates one’s perceived social network or support received from family and friends.
- Functioning, measured by World Health Organization Disability Assessment Schedule 2.0 (WHODAS 2.0), evaluates an individual’s health-related functioning and disability across 6 life domains within the International Classification of Functioning, Disability and Health (ICF) framework [34]. WHODAS 2.0 is a cross-culturally valid tool that reflects a person’s ability to participate in daily life activities (personal perspective: cognition, mobility, and self-care) and to engage with their environment (social perspective: getting along, life activities, and participation).
- Social engagement, determined through a subjective assessment using the 6-item Index of Social Engagement (ISE), which describes an individual’s self-evaluation of both social involvement and autonomy, is used to capture subjective social engagement [43], and objective evaluations via the 10-item Social Engagement and Activities Questionnaire (SEAQ).

**Secondary outcomes**

- ***Hearing health outcomes***
  - Hearing handicap is measured by the Hearing Handicap Inventory Screening Questionnaire for Adults (HHIA). Participants’ self-reported hearing status is also collected to reflect subjective hearing impairment (HI). For the hearing aid use (HAU) group, the International Outcome Inventory for Hearing Aids (IOI-HA) will be used during the follow-up period to verify the treatment effects of HAU.
  - Hearing-related health literacy is measured through a set of 9 questions developed by the research team based on the Knowledge, Attitude, and Practice theory. The 9-item assessment evaluates the respondents’ understanding (eg, information on hearing health, preventive measures, and awareness of available hearing health care), attitudes (eg, beliefs and attitudes toward hearing health care, willingness to prioritize and engage in hearing health activities), and behaviors (eg, regular hearing check-ups, protection from loud noises, the use of hearing aids if needed) related to hearing health [44]. The content and validity of the 9-item questionnaire were evaluated by 5 experts in hearing and health education.
  - Communication ability is measured in the follow-up survey using the 10-item Self-Assessment of Communication (SAC), a self-report questionnaire designed to assess the impact of hearing loss and hearing intervention outcomes [45].
- ***Psychosocial and health-related outcomes***
  - Self-efficacy is assessed by the 10-item Chinese version of the General Self-Efficacy Scale (GSE). It has been identified as a good tool for measuring self-efficacy in community-dwelling adults [46].
  - A sense of mastery is measured by the 7-item Chinese version Pearlin Mastery Scale (PMS). In addition to self-efficacy, mastery serves as another psychological resource that may help mitigate the impact of stressors on well-being. PMS has been regarded as a well-validated measure commonly used in studies to examine adults’ health and well-being outcomes [47].
  - Psychological resilience in the HHE group is evaluated using the 2-item Connor-Davidson Resilience Scale (CD-RISC). The 2-item CD-RISC has demonstrated good validity and reliability in the elderly or Chinese general population [48].
  - Quality of life includes both a subjective and an objective measure. Health-related quality of life is measured by standard tests such as the EQ-5D-5L, and subjective well-being is measured by the 5-item Chinese version of the Satisfaction with Life Scale (SWLS).

### Covariates

Sociodemographic factors (age, sex, residency, marital status, education, income status, and employment), lifestyle behaviors (smoking and drinking), and health conditions (visual status, diagnosed with hypertension, diabetes, or any other cardiovascular diseases, and self-reported health) of the eligible participants are collected during the baseline survey.

### Statistical Analysis

The intent-to-treat principle will be used during the impact analysis. The differences between the control and intervention groups in terms of primary and secondary outcomes will be evaluated through ANOVA and linear mixed-effects modeling. Multiple methods such as weighting, matching, and imputation will be used to handle the problem of missing data and sample attrition. In addition, a nonparametric bias-corrected case resampling bootstrap method, structural equation modeling, or interaction analysis will be used to further explore the buffering effect of psychosocial factors. Heterogeneity analysis will be applied to investigate whether the intervention effects are robust across subgroup samples, such as participants with different levels of objective hearing loss.

The cost-effectiveness analysis will be based on the costs of the HAU or HHE interventions and their effects on improving health-related quality of life, as measured by EQ-5D-5L. EQ-5D-5L is designed to measure the generic quality of life in terms of mobility, self-care, usual activities, pain or discomfort, and anxiety or depression [49]. Previous literature has used this multidimensional construct of quality of life to evaluate the effect of hearing interventions [50-53].

### Data Collection and Management

Data collection forms and procedures for data management are available upon request. All the data will be collected through a structured paper questionnaire. Trained enumerators will conduct face-to-face interviews with each eligible participant and complete the forms. Double-blind data entry will be performed to input the baseline and follow-up data into electronic form for quality assurance purposes. All information collected during the trial will be kept in strict confidence. Participant details will be stored on a secure, password-protected computer disk and mobile hard disk drive. The confidentiality of the intervention objectives during qualitative interviews will be safeguarded by assigning a unique identification code to electronic sound files and transcripts of the interviews. The information will be known only to the qualitative researcher and appropriate members of the research team. The principal investigator (PI) and data analysis personnel will have access to the final trial dataset. According to the Good Clinical Practice guidelines, data will be archived for a period of 10 years after finalizing the study with participants’ informed consent. To enhance and protect participant confidentiality, any quotes published will be anonymous. After finalization, the key file containing information linking participants’ names and contact details will be destroyed once the project team no longer needs to contact the participants further for study purposes.

### Monitoring

#### Data Monitoring

The study is subject to local regulations. The principal investigator is responsible for developing, implementing, and maintaining the quality assurance and control of all the research. During the period of sample recruitment, an interim analysis will be conducted under strict confidentially, which may include analyses of data from other comparable trials. Through these interim analyses, the PI will determine if the interventions or potential mechanisms have been proven or if they differ from the expectations. The PI will decide whether to modify the trial. Unless this situation occurs, the research staff will remain unknown during the interim analysis.

#### Adverse Events

Adverse events will be collected and recorded after informed consent and this procedure will continue until the end of the study. For those who report an adverse event after submitting the signed informed consent but before the trial intervention, the event will not be considered as an adverse event related to our intervention. Serious adverse events will be reported to the ethics review committee.

#### Auditing and Inspecting

The PI will allow study-related audits and inspections by the ethics review committee of the documents pertaining to the research. The PI will guarantee the capability to conduct inspections of applicable study-related facilities.

### Ethical Considerations

The study was approved by the Chinese Ethics Committee of Registering Clinical Trials (ChiECRCT, Reference number: ChiECRCT20210561). The trial is registered on ClinicalTrials.gov (registration number: ChiCTR2200062148; date of registration: July 25, 2022). If there are any changes to the protocol, we will report to the ChiECRCT and inform the subjects. A formal amendment to the protocol will be submitted to and approved by the ethics review committee before implementation.

During the enrollment session, participants receive detailed information about the study and are required to provide written informed consent before undergoing the auditory examination. Regardless of their inclusion in subsequent studies, participants received compensation consisting of 30 Yuan (US $4.2) in cash and a gift of daily necessities for their participation in the hearing tests. In the follow-up survey, respondents will also receive 30 yuan (US $4.2) in cash after completing the questionnaire.

Each respondent is assigned a personal identification number, which serves as a unique identifier for correlating the participants’ identification details with information related to the trial, thereby safeguarding confidentiality. No further identifiable features will be included in the data analysis process.

## Results

### Trial Status

**Figure 2:**
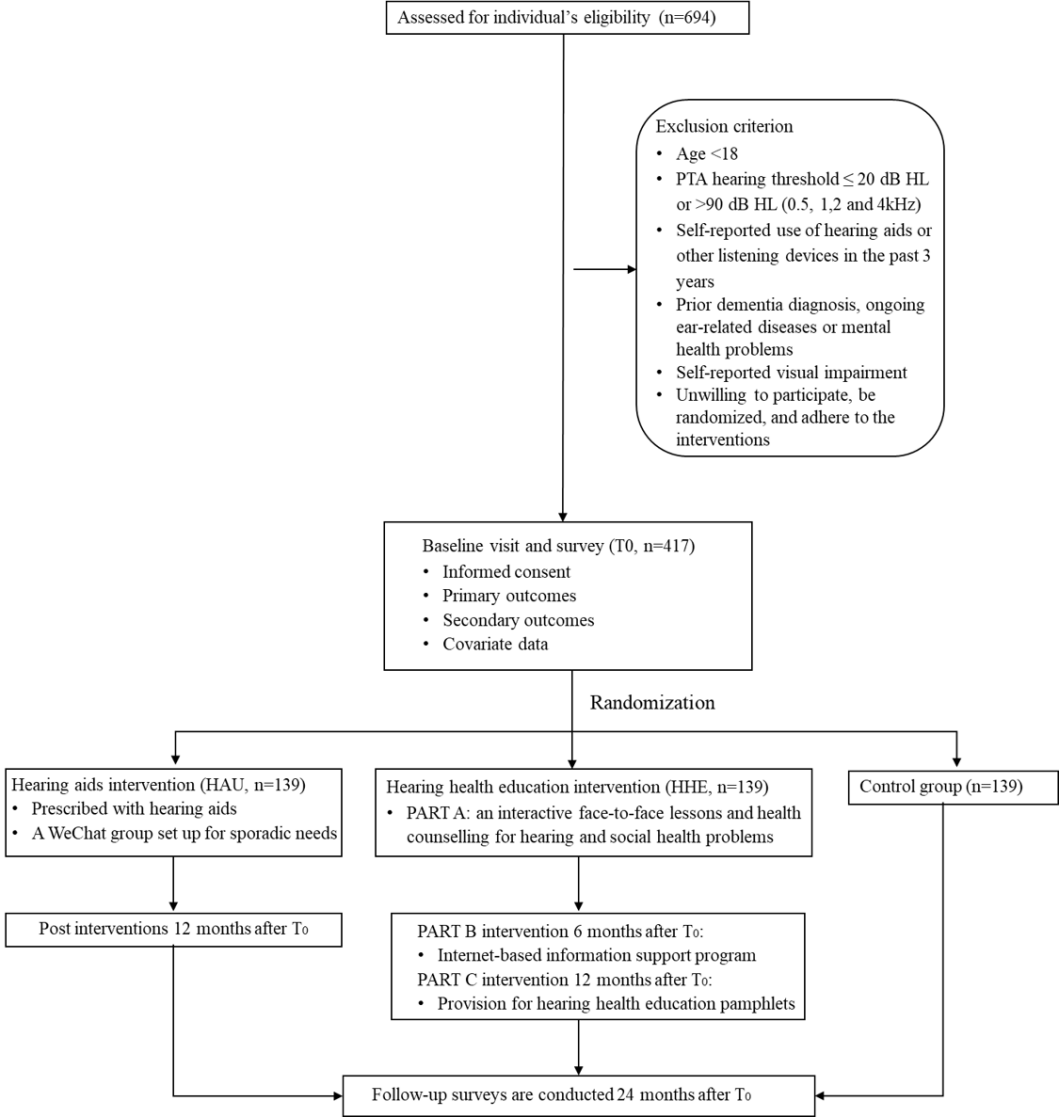
Hearing Impairment and Social Outcomes Evaluation in Adults study flow diagram developed using Standard Protocol Items for Interventional Trials or Consolidated Standards of Reporting Trials.

The project was funded in July 2019. The current protocol is version 3, dated January 15, 2025. The recruitment of participants and collection of baseline data began on September 1, 2022, and ended on September 30, 2022. A total of 435 participants were recruited and assigned to a random group at baseline. A follow-up survey was conducted after 24 months in September 2024, which included 397 participants. Figure 2 displays the flow diagram of the HISEA trial. Until this version of the protocol, the intervention effects have remained unknown.

### Preliminary Results

Table 2 shows the sociodemographic characteristics of the sample at baseline. The mean (SD) age of the participants in RCT is 71.59 (SD 9.55) years, with 43.09% (187/434) of the sample identifying as female.

**Table 2:** Baseline sample characteristics.

Variables		Control group	Hearing health education group	Hearing aid use group	Total
Age (years), mean (SD)		74.82 (9.29)	71.44 (6.51)	67.02 (11.75)	71.59 (9.55)
**Sex, n (%)**					
	Male	56 (35.9)	56 (35.67)	75 (61.98)	247 (56.91)
	Female	100 (64.1)	101 (64.33)	46 (38.02)	187 (43.09)
**Education, n (%)**					
	Illiterate	43 (27.74)	27 (17.2)	30 (25.21)	100 (23.2)
	Primary school	86 (55.48)	94 (59.87)	61 (51.26)	241 (55.92)
	Middle school or higher	26 (16.77)	36 (22.93)	28 (23.53)	90 (20.88)
**Marital status, n (%)**					
	With spouse	116 (75.32)	95 (67.14)	88 (76.52)	298 (72.86)
	No spouse	38 (24.68)	46 (32.86)	27 (23.48)	111 (27.14)
**Self-rated economic status, n (%)**					
	Fair	115 (74.68)	109 (70.32)	24 (20)	248 (57.81)
	Poor	39 (25.32)	46 (29.68)	96 (80)	181 (42.19)
**Self-rated health, n (%)**					
	Good	76 (48.72)	149 (94.9)	52 (42.98)	277 (63.82)
	Fair	65 (41.67)	7 (4.46)	46 (38.02)	118 (27.19)
	Poor	15 (9.62)	1 (0.64)	23 (19.01)	39 (8.99)
**Chronic disease, n (%)**					
	No	22 (20)	39 (27.08)	70 (61.4)	131 (35.6)
	Yes	88 (80)	105 (72.92)	44 (38.6)	237 (64.4)
Number of observations, n		156	158	121	435

## Discussion

### Principal Findings

We anticipate a significant positive effect of interventions on reducing social isolation and enhancing functioning outcomes, along with several psychological pathways, which contribute to these main results.

### Comparison to Previous Work

This RCT assesses the impact of 2 interventions—the provision of free hearing aids and an HHE program on social connections and functioning among community-dwelling Chinese adults with HI. The design of this RCT is partly similar to the Aging and Cognitive Health Evaluation in Elders (ACHIEVE) RCT conducted in the United States, which commenced in 2017. The ACHIEVE trial implemented a 3-year hearing intervention that included audiological counseling and provision of hearing aids, alongside a control intervention on successful aging health education. A notable distinction in our study’s design is the inclusion of a control group that receives no intervention, as well as the specificity of our health education to hearing-related topics. While the ACHIEVE trial primarily targeted cognitive outcomes, it also assessed some mental health and well-being outcomes [54]. In contrast, our study is specifically oriented towards social functioning. Furthermore, secondary analysis of ACHIEVE data indicated no significant association between the 3-year interventions—either hearing aid provision or health education—and health-related quality of life [55]. Our research will help examine the effectiveness of these interventions among older adults with HI within a different sociocultural context. Our use of WHO-DAS II as a generic measure in the hearing-impaired population is similar to another 12-month hearing aid intervention research conducted in the United States [23].

Currently, among the completed RCTs concerning community hearing intervention rehabilitation in China, 2 studies exhibit similarities to our community interventions. One of these studies conducted a 20-month hearing aid intervention [51], while the other implemented a 3-month hearing aid combined with a health education intervention [56]. Both studies used a 2-arm design and assessed a range of outcome variables, with secondary outcomes including quality of life and social isolation. Our intervention design is characterized by a more complex 3-arm intervention design and a larger sample size, which is anticipated to yield more robust evidence regarding the long-term effects of hearing interventions. To our knowledge, this will be the first RCT to evaluate the efficacy of community-delivered hearing rehabilitative and supportive interventions—both hearing aids and health education—on older adults’ SWB in the context of Chinese aging society.

### Strengths

This study highlights 2 main strengths. First, the study makes a theoretical contribution by integrating the WHO’s ICF and IPC-EHC frameworks in conjunction with the stress process model. The WHO frameworks facilitate a focus on both individual and environmental factors in the design of interventions, emphasizing social isolation and functional outcomes, which distinguishes this research from traditional studies that primarily address quality of life and cognitive functions. Within the context of the stress process model, hearing deterioration is conceptualized as a chronic stressor that can have both direct and indirect effects on psychosocial distress [57]. Thus, the psychological pathway outlined in our hearing intervention aims to provide empirical support for the application of the stress model in the management of chronic diseases such as hearing loss, offering a detailed explanation of the role of stress within the model in relation to different health consequences.

Second, the interventions are community-oriented, which aligns with China’s policy setting on hearing rehabilitation and health promotion. Currently, China’s Action for Promoting Hearing Health in the Elderly mainly conducts activities based on communities, such as popularizing knowledge about hearing health, screening, and public health initiatives. Besides, both online and offline elements are blended in the interventions, which provides more convenient and efficient support and meets with current digital health trends. The findings of this study may provide valuable insights for health care and social workers to enhance well-being among older adults through auditory interventions.

### Limitations

Despite these strengths, the trial faces several limitations. First, the design of a one-time follow-up study presents challenges in evaluating the short-term effects of interventions, as well as their impacts beyond a 24-month period. The duration of intervention and follow-up is selected based on the following considerations: (1) previous research has consistently shown significant findings regarding the short-term effects of hearing aid interventions; however, the medium- to long-term effects remain ambiguous, and adherence to the prolonged use of hearing aids is notably low. Our primary interest lies in investigating these medium- to long-term effects; (2) given that the measurement tools used in both the baseline and follow-up questionnaires are fixed, we intend to extend the interval between the 2 measurements to mitigate potential learning effects; and (3) in addition, the constraints imposed by the project’s duration and funding are considered.

Second, there is a lack of subjective measures in our survey questionnaire. To address this gap, we conducted qualitative interviews after the RCT. A subset of older adults with HI from intervention groups were inquired about their satisfaction with the use of hearing aids, their experience in health education activities, and their assessments of the health education materials. In addition, we gathered feedback from CPs.

Third, the provision of free hearing aids may raise concerns regarding the social approval effect. Previous research on hearing aid interventions has typically involved the distribution of free devices [51,58,59]. In instances where there is a financial burden associated with acquiring commercial hearing aids, selection bias may arise, as economically disadvantaged older individuals may find it more challenging to participate in such interventions. The Chinese government has established a range of welfare policies aimed at supporting individuals with disabilities and the elderly, which include not only rehabilitation services, but also living subsidies for disabled individuals, long-term care insurance, and older adult care subsidies. Within this welfare framework, hearing aids do not represent a unique benefit. Therefore, we contend that the social approval effect remains within a manageable scope and is unlikely to compromise the reliability and validity of the RCT. Future research could also compare the intervention effects of paid hearing aids with those of free hearing aids.

Fourth, the interventions are conducted at different sites, which may result in geographical and selection biases. Nevertheless, the comparable socioeconomic and cultural contexts of these sites are expected to alleviate such biases. There was no pilot study due to time and budget constraints; however, given that the social and demographic characteristics of the feasibility study site closely resemble those of the formal trial sites, we believe this limitation does not pose a significant threat to the research design.

Fifth, our reliance on self-reported measures of social isolation and functioning may introduce measurement bias, which we will address through the use of validated tools, cross-validation, and multisource evaluations.

Lastly, although the sample size was determined using standard calculation methods, it remains relatively small, which affects its representativeness and external validity. Randomization in a small sample may not completely eliminate the preexisting differences either. Therefore, we will further use matching techniques or other econometric methods during the statistical analyses.

### Dissemination Plan

The results of the study will be disseminated through peer-reviewed publications and national and international conference presentations to publicize and explain the research to key audiences. Dissemination authorship will be fulfilled by the research team’s efforts. Information regarding public access can be seen on the Chinese Clinical Trial Registry website.

### Future Directions

This trial is poised to provide early insights into the feasibility, acceptability, and effectiveness of community-delivered rehabilitation interventions aimed at mitigating the social impacts of HI. The findings will inform future improvements in hearing health care and community-based health practices.

### Conclusions

The findings of this study will evaluate the impact and cost-effectiveness of a community-based auditory or hearing health education intervention on SWB among Chinese adults with HI. It will help promote hearing health in older adults and decrease adverse health risks in an aging society.

## Appendix

Multimedia Appendix 1 SPIRIT (Standard Protocol Items for Interventional Trials) checklist.

Multimedia Appendix 2 CONSORT-eHEALTH checklist (V1.6.1).

## Abbreviations

ACHIEVE: Aging and Cognitive Health Evaluation in Elders
CDPF: China Disabled Persons’ Federation
ChiECRCT: Chinese Ethics Committee of Registering Clinical Trials
CP: communication partner
GPSRC: Guangdong Provincial Cultural Sports and Rehabilitation Assistive Devices Center for People with Disabilities
HAU: hearing aid use
HHE: hearing health education
HI: hearing impairment
HISEA: Hearing Impairment and Social Outcomes Evaluation in Adults
ICF: International Classification of Functioning, Disability and Health
IPC-EHC: integrated person-centered ear and hearing care
LSNS: Lubben Social Network Scale
PI: principal investigator
RCT: randomized controlled trial
SPIRIT: Standard Protocol Items for Interventional Trials
SWB: social well-being
WHO: World Health Organization
WHODAS 2.0: World Health Organization Disability Assessment Schedule 2.0

## Data Availability

The datasets generated and analyzed during this study are not publicly available due to the confidentiality agreement reached with the participants but are available from the corresponding author on reasonable request.
